# Supplementary material for: Id2 Represses Aldosterone-Stimulated Cardiac T-Type Calcium Channels Expression
Source: Int J Mol Sci. 2021 Mar 30;22(7):3561. doi: 10.3390/ijms22073561 (PMC8037527; doi:10.3390/ijms22073561)
Supplement: Supplementary file 1 [file ijms-22-03561-s001.pdf]

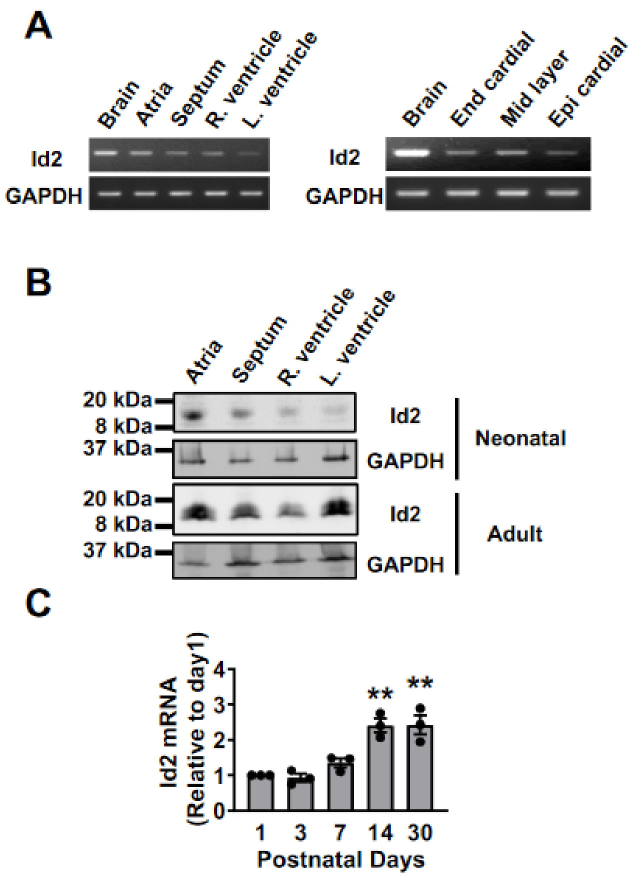


**<u>Figure S1.</u> Id2 expression the heart tissues of rats.** (A) Pictures show the RT-PCR analysis of Id2 (upper) and GAPDH (lower) mRNA expression in brain and adult cardiac tissue: atria, septum, right (R) ventricle, and left (L.) ventricle (left); and brain and left ventricular free wall (right): End, endocardial layer; Mid, mid-myocardial layer; Epi, epicardial (Epi) layer. Pictures are representative of 3 individual experiments (B) Pictures are western blot experiments showing the expression of Id2 and GAPDH in neonatal (top) and adult (bottom) cardio tissue. Pictures are representative of 3 individual experiments (C) Graph are the mean expression of Id2 mRNA in the whole-heart of rat measured by RT-qPCR 1, 3, 7, 14, and 30 days after birth. Bars and error bars indicate the mean + s.e.m. (n = 3). ***P < 0.001 compared to day 1.

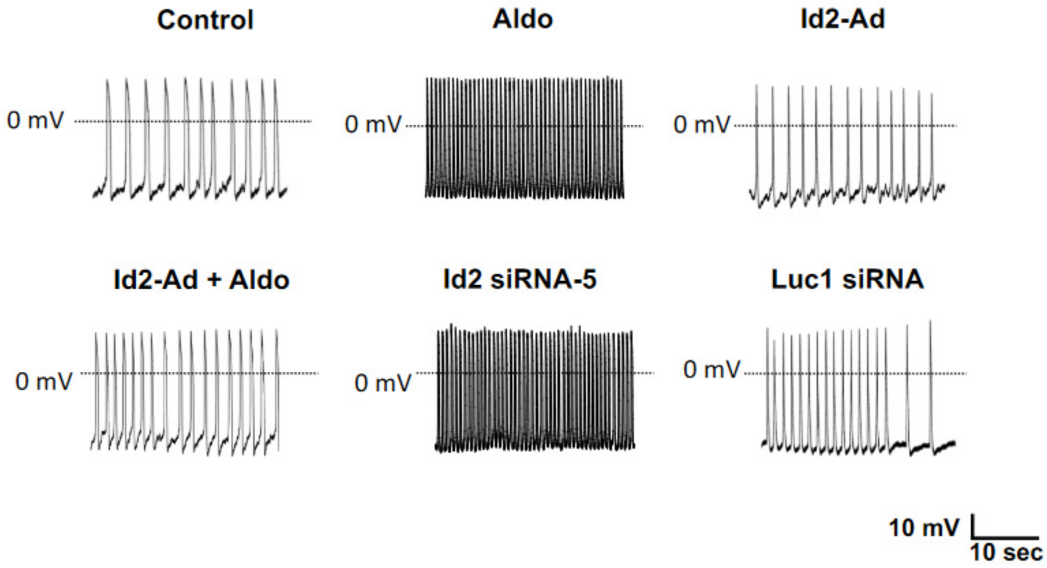


**<u>Figure S2.</u> Spontaneous action potential recordings of neonatal rat cardiomyocytes** Graphs are 30 seconds of spontaneous action potentials recorded by patch-clamp in isolated neonatal ventricular cardiomyocytes. Each graph is a representative spontaneous action potential recorded in a single control, aldosterone-stimulated control, Id2-overexpressing, aldosterone-stimulated Id2-overexpressing, Id2 siRNA-treated, and Luciferase siRNA-treated cardiomyocytes.

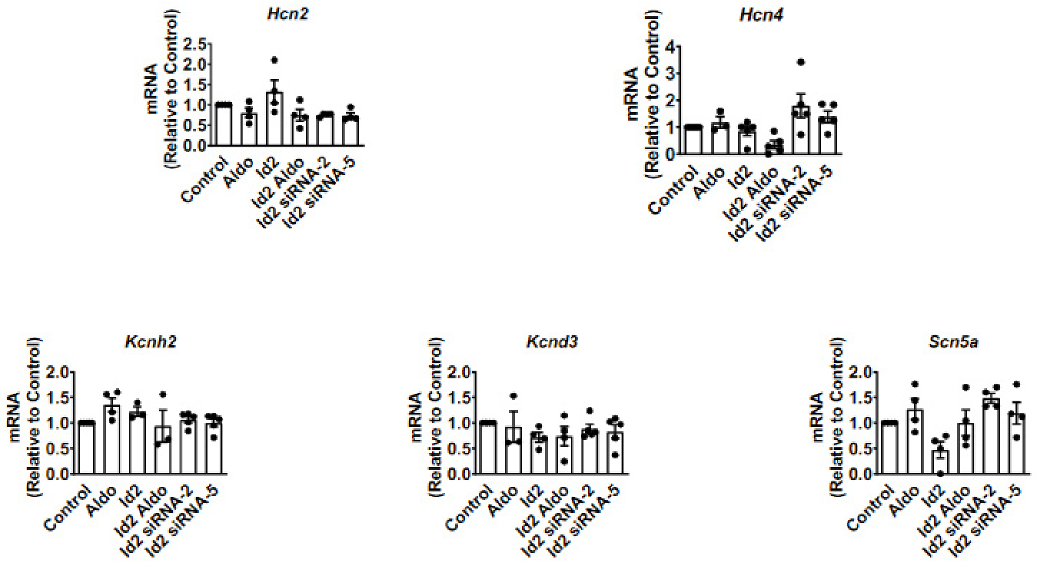


**<u>Figure S3.</u> Expression various cardiac channels expression in neonatal rat ventricular cardiomyocytes.** The expression of *Hcn2*, *Hcn4*, *Kcnh2*, *Kcnd3, and Scn5a* mRNA were measured by RT-qPCR in control, aldosterone-treated, Id2 overexpressing, aldosterone-treated Id2 overexpressing, Id2 siRNA treated neonatal rat cardiomyocytes. Graphs are the mean + s.e.m of n=3-5.

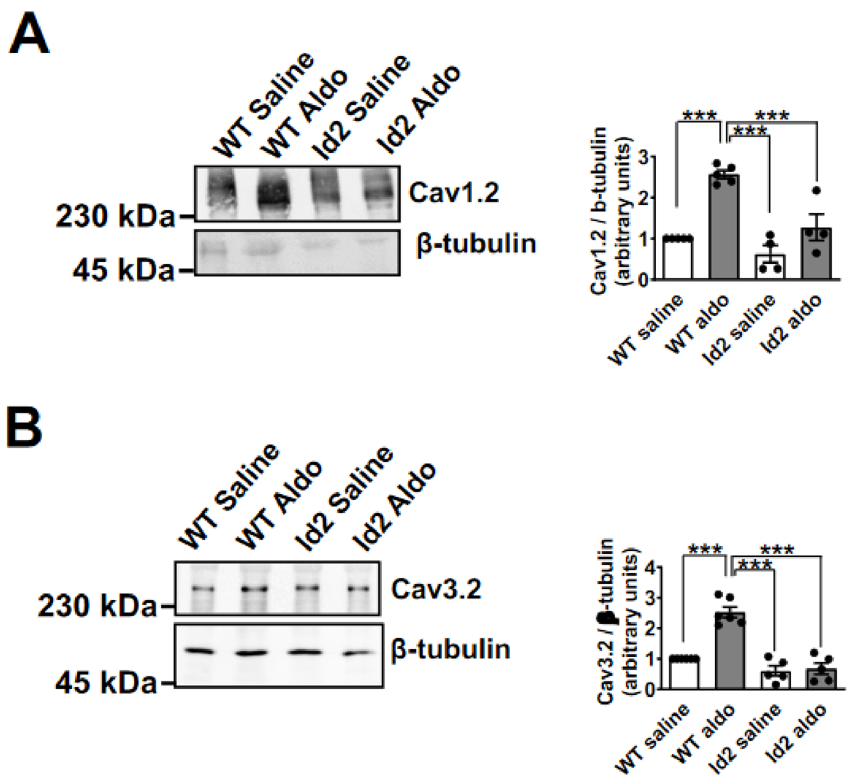


**Figure S4.** Id2 expressing transgenic mice prevents the aldosterone-stimulated expression of CaV1.2 and CaV3.2 voltage-gated calcium channels *in vivo*. Pictures of western blotting experiments showing (A) CaV1.2 and (B) CaV3.2 (upper) and tubulin (lower) proteins expression levels in WT or Id2 transgenic mice treated with saline solution or aldosterone. Left Graph is are the mean expression of CaV1.2 (A), CaV3.2 (B) expression (n=4 for CaV1.2 and n=5 for CaV3.2). Bars and error bars indicate the mean + s.e.m., ****P* < 0.001.

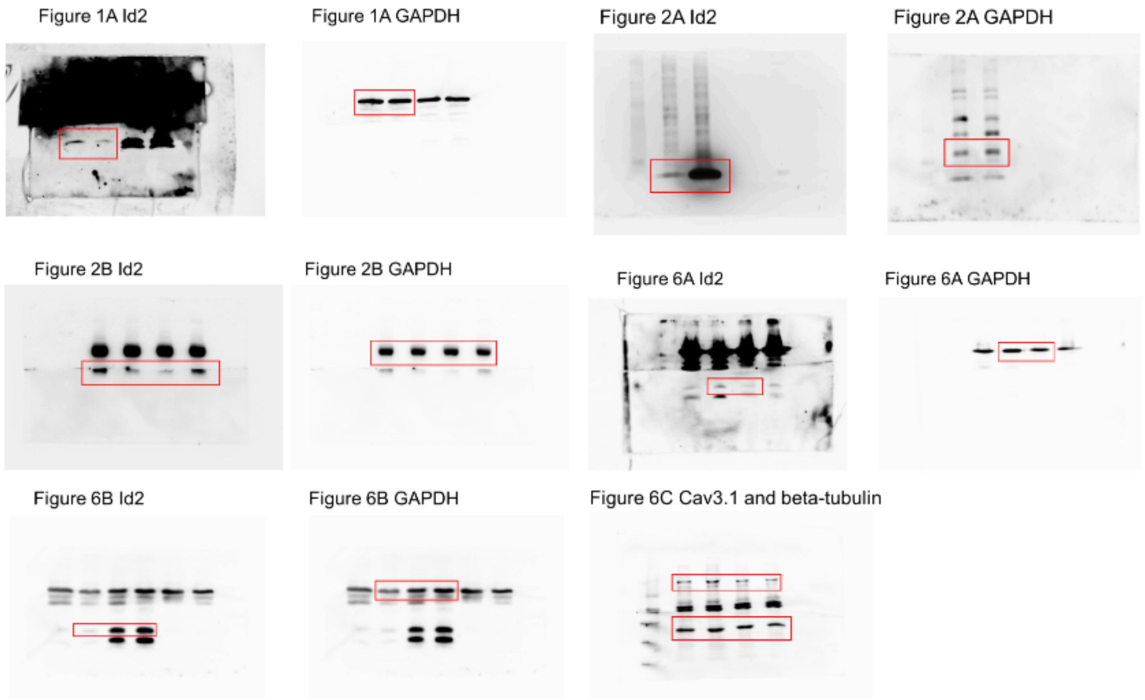


**Figure S5. Whole pictures of western blots experiments.** Pictures are whole pictures of western blots experiments shown in figure 2 and 6.

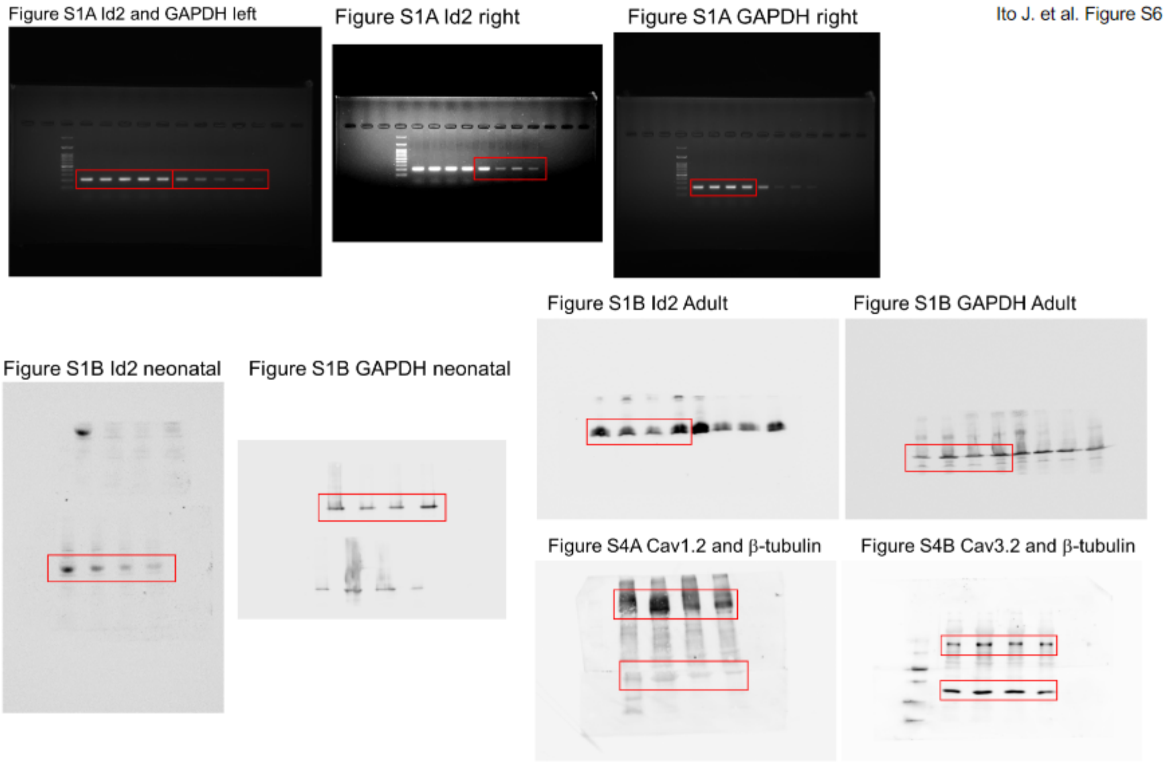


**<u>Figure S6.</u> Whole pictures of western blots and RT-PCR experiments.** Pictures are whole pictures of western blots experiments shown and RT-PCR experiments in figure S1 and S4.
